# The efficacy of vigorous-intensity exercise as an aid to smoking cessation in adults with elevated anxiety sensitivity: study protocol for a randomized controlled trial

**DOI:** 10.1186/1745-6215-13-207

**Published:** 2012-11-13

**Authors:** Jasper A J Smits, Michael J Zvolensky, David Rosenfield, Bess H Marcus, Timothy S Church, Georita M Frierson, Mark B Powers, Michael W Otto, Michelle L Davis, Lindsey B DeBoer, Nicole F Briceno

**Affiliations:** 1 Department of Psychology, Southern Methodist University, Dedman College, P.O. Box 750442, Dallas, TX, 75275, USA; 2 Department of Psychology, University of Houston, Houston, TX, USA; 3 Department of Family & Preventive Medicine, University of California, San Diego, CA, USA; 4 Pennington Biomedical Research Center, Louisiana State University, Baton Rouge, LA, USA; 5 Department of Psychology, Howard University, Washington, DC, USA; 6 Department of Psychology, Boston University, Boston, MA, USA

**Keywords:** Smoking, Smoking cessation, Intervention, Randomized controlled trial, Exercise, Aerobic exercise, Anxiety, Anxiety sensitivity

## Abstract

**Background:**

Although cigarette smoking is a leading cause of death and disability in the United States (US), over 40 million adults in the US currently smoke. Quitting smoking is particularly difficult for smokers with certain types of psychological vulnerability. Researchers have frequently called attention to the relation between smoking and anxiety-related states and disorders, and evidence suggests that panic and related anxiety vulnerability factors, specifically anxiety sensitivity (AS or fear of somatic arousal), negatively impact cessation. Accordingly, there is merit to targeting AS among smokers to improve cessation outcome. Aerobic exercise has emerged as a promising aid for smoking cessation for this high-risk (for relapse) group because exercise can effectively reduce AS and other factors predicting smoking relapse (for example, withdrawal, depressed mood, anxiety), and it has shown initial efficacy for smoking cessation. The current manuscript presents the rationale, study design and procedures, and design considerations of the Smoking Termination Enhancement Project (STEP).

**Methods:**

STEP is a randomized clinical trial that compares a vigorous-intensity exercise intervention to a health and wellness education intervention as an aid for smoking cessation in adults with elevated AS. One hundred and fifty eligible participants will receive standard treatment (ST) for smoking cessation that includes cognitive behavioral therapy (CBT) and nicotine replacement therapy (NRT). In addition, participants will be randomly assigned to either an exercise intervention (ST+EX) or a health and wellness education intervention (ST+CTRL). Participants in both arms will meet 3 times a week for 15 weeks, receiving CBT once a week for the first 7 weeks, and 3 supervised exercise or health and wellness education sessions (depending on randomization) per week for the full 15-week intervention. Participants will be asked to set a quit date for 6 weeks after the baseline visit, and smoking cessation outcomes as well as putative mediator variables will be measured up to 6 months following the quit date.

**Discussion:**

The primary objective of STEP is to evaluate whether vigorous-intensity exercise can aid smoking cessation in anxiety vulnerable adults. If effective, the use of vigorous-intensity exercise as a component of smoking cessation interventions would have a significant public health impact. Specifically, in addition to improving smoking cessation treatment outcome, exercise is expected to offer benefits to overall health, which may be particularly important for smokers. The study is also designed to test putative mediators of the intervention effects and therefore has the potential to advance the understanding of exercise-anxiety-smoking relations and guide future research on this topic.

**Clinical trials registry:**

ClinicalTrials.gov, NCT01065506, http://clinicaltrials.gov/ct2/show/NCT01065506

## Background

### Tobacco use: scope of problem

Cigarette smoking is a leading cause of death in the United States (US), contributing to over 440,000 deaths each year [1]. Cigarette smoking is implicated in various types of medical illness, including heart disease, pulmonary diseases, and cancer [2]. Importantly, quitting smoking decreases the risk of developing medical problems and may increase survival time among those persons who have already developed medical problems [3]. However, despite a substantial decline in smoking prevalence since the 1960s, smoking remains prevalent, with a recent report estimating that 20.8% of US adults are current cigarette smokers [4]. Reports further indicate that although most smokers (70%) are motivated to quit [5], cessation failure (that is, relapse) is high among smokers who try to quit smoking on their own (90% to 95% [6]) or attend treatment programs (60% to 80% [7,8]). In fact, since the mid 1970s, abstinence rates associated with standard behavioral pharmacological therapies have decreased [9].

One response to these observations has been a call for the development of specialized or targeted interventions that address specific needs of smokers who are at higher risk for relapse [10,11]. Guiding this direction is the selection/hardening hypothesis of smoking prevalence, which posits that smokers who are unable to quit successfully are ‘burdened’ by factors that pose particular challenges to quitting [12-14]. According to this perspective, continuing smokers are mostly at-risk smokers who cannot quit, thus explaining why smoking prevalence has begun to stabilize over the years.

### Smoking and anxiety and its disorders

Anxiety psychopathology co-occurs with smoking at rates that exceed those found in non-psychiatric populations [15]. Reported rates of smoking are highest among individuals with panic-related problems and other anxiety disorders where panic attacks are particularly common (for example, social anxiety disorder, posttraumatic stress disorder [PTSD], generalized anxiety disorder [16-20]). Moreover, the observed association between smoking and anxiety psychopathology is not due to sociodemographic characteristics, other psychiatric co-morbidities, or symptom overlap in diagnostic criteria for anxiety disorders and nicotine dependence [21]. Numerous studies indicate anxiety disorders significantly impair cessation success [16,22,23]. For example, Piper and colleagues [24] examined the relation of psychiatric disorders to tobacco dependence and cessation outcomes among 1,504 people making a pharmacologically-aided smoking cessation attempt as part of a clinical trial. Six months after quitting, those with an anxiety disorder had the lowest abstinence rates.

### Anxiety sensitivity and smoking

In order to isolate possible therapeutic targets and transdiagnostic mechanisms for treatment development, we and others have explored numerous factors that could account for why anxiety is often associated with smoking relapse (for example, severity of nicotine dependence, age of smoking onset, broad-based tendency to experience negative mood). Here, some of the strongest and most consistent evidence has been evident for anxiety sensitivity (AS) [25]. AS is defined as the fear of anxiety or related sensations (for example, racing heart, chest pain, rapid breathing, dizziness). This fear is often fueled by concerns about physical (for example, heart attack, stroke, death), social (for example, embarrassment), or mental (for example, going crazy, losing control) catastrophes. Historically, AS has been studied to better understand the etiology and maintenance of anxiety and its disorders, particularly panic disorder and PTSD [26-33]. More recent work suggests that AS also plays a formative role in smoking behavior. For example, AS is positively correlated with smoking to reduce negative affect, but often not with other smoking motives (for example, handling, taste [34-39]). Other studies have found that AS is related to negative affect reduction expectancies for smoking (beliefs that smoking will reduce negative affect [40,41]). Additionally, smokers high in AS perceive the prospect of quitting as both a more difficult and personally threatening experience [42], possibly due to a hypersensitivity to aversive internal sensations such as nicotine withdrawal symptoms [43] or elevated state anxiety [44-46]. High AS smokers compared to those low in AS also experience greater increases in positive affect from pre- to post-cigarette consumption and report greater smoking satisfaction [47]. Perhaps most notably, AS is significantly associated with less success during smoking cessation attempts [48]. Specifically, higher levels of AS are related to greater odds of early lapse [40] and relapse during quit attempts [49-51]; these effects are not explained by smoking rate or nicotine dependence, nicotine withdrawal symptoms, or trait-like negative mood propensity [50].

Collectively, these studies suggest that AS is associated with problems during cessation and is correlated with smoking to reduce negative affect. Moreover, AS moderates the risk of smoking in terms of the development of panic attacks, suggesting that regular smokers with higher AS are at increased risk for experiencing panic-related problems [52]. Unlike many other panic risk factors (for example, family history of psychiatric illness), AS is malleable in response to exposure-based intervention and can therefore be specifically targeted for therapeutic change. As we now discuss, exercise is one method for reducing AS and thereby promoting abstinence for this high-risk group.

### Exercise as an intervention for reducing AS and promoting smoking abstinence

There are several findings that support studying the efficacy of exercise as an aid to smoking cessation among smokers with elevated AS. First, exercise has been shown to significantly reduce AS [53,54], perhaps through repeated exposure to feared bodily sensations (for example, sweating, rapid heartbeat [55]). Second, exercise has been shown to reduce nicotine withdrawal symptoms [56,57], negative affect [56], and depressed mood and anxiety [55,58,59], all of which predict cessation relapse, particularly among persons with elevated AS [50]. Third, there is evidence [60] of the efficacy of exercise as an effective smoking cessation intervention when it is supervised and uses an intense dose (for example, vigorous-intensity, three times per week over 12 weeks). This particular trial [60] involved the randomization of sedentary female smokers to either a 12-week cognitive-behavioral smoking cessation program with vigorous-intensity exercise (three sessions a week of 30 to 40 min at 60% to 85% of heart rate reserve), or a 12-week cognitive-behavioral smoking cessation program with contact control (three 45 to 60 min health education sessions a week). All participants initiated the intervention 3 weeks prior to the quit date of the smoking cessation program. The results revealed that participants assigned to the exercise condition were more likely than participants assigned to the control intervention to be continuously abstinent during the 8 weeks (19.4% *vs*. 10.2%), 20 weeks (16.4% *vs*. 8.2%), and 60 weeks (11.9% *vs*. 5.4%) following the quit day.

Although existing work on exercise and smoking cessation is indeed promising (see [57] for a review), it cannot speak to the utility of exercise as an aid for smoking cessation among individuals with elevated AS. Indeed, previous exercise-smoking cessation studies have tended to exclude for psychiatric vulnerability or disorders [57]. These exclusion criteria may preclude generalizing the studies’ findings to at risk populations such as high AS smokers, a population who may arguably benefit most from such interventions. Second, exercise studies conducted to date did not advance the knowledge of the mechanisms by which exercise facilitates the efficacy of standard smoking cessation protocols. Thus, there are no data on the potential mediating effects of reductions in AS, nicotine withdrawal symptoms, depressed mood, and anxiety on reductions in smoking. Third, many exercise studies conducted to date have focused exclusively on women [57]. Accordingly, there is little empirical information about the utility of exercise for smoking cessation for men. Although exercise effects are likely to benefit men and women [54], marshalling empirical data across sex is an important task for exercise-smoking oriented work.

### Study aims

The primary aim of STEP is to compare the relative effects of two smoking cessations interventions (standard treatment combined with exercise (ST+EX) *vs*. standard treatment combined with a wellness education intervention (ST+CTRL)) on three central smoking cessation outcomes: (1) point prevalence abstinence (PPA); (2) time to first smoking lapse; and (3) time to first smoking relapse. Smoking will be measured by self-report using the time-line follow back (TLFB) procedure and verified by carbon monoxide and cotinine assessment (see Assessments below). We hypothesize that PPA will be higher, both in the short term and long term, for those in the ST+EX condition than for those in the ST+CTRL condition. Similarly, we expect the rate of decline in abstinence over time to be shallower (smaller) in ST+EX than in ST+CTRL. Lastly, we expect mean time to first lapse and to relapse to be greater for those in the ST+EX compared to those in the ST+CTRL condition.

**Figure 1:**
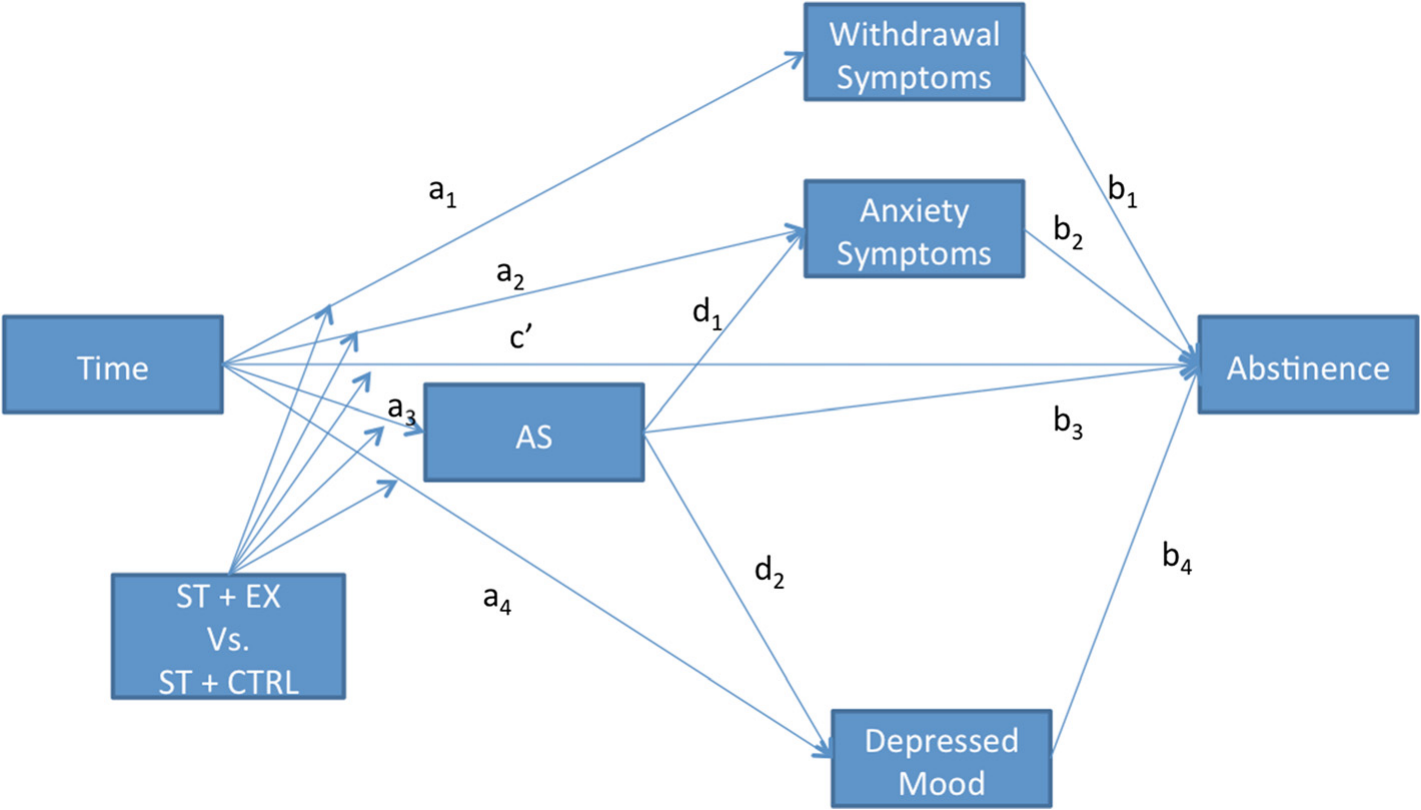
Model of study aims and hypotheses.

The secondary aim of STEP is to investigate the putative mechanisms by which exercise increases smoking cessation outcomes. Figure 1 illustrates the central predictions of our model of the mechanisms of change in abstinence. We expect that: (1) treatments over time will directly cause changes in abstinence and in all proposed mediators of change in abstinence (as indicated by the ‘a’ paths in Figure 1 linking time to withdrawal symptoms, AS, anxiety symptoms, depressed mood, and abstinence); (2) the effect of treatment over time on all of these variables will be moderated by treatment condition, such that those receiving ST+EX will improve more than those receiving ST+CTRL; (3) changes in each mediator over time will lead to improvements in abstinence rates over time (as indicated by the ‘b’ paths in Figure 1); and Table 1 decreases in AS over time will lead to improvements in anxiety symptoms and depressed mood over time (‘d’ paths in Figure 1).

**Table 1:** Overview of assessments

	**Protocol weeks**								
	**>−3**	**−2**	**−1**	**0**	**1**-**5**	**6**	**8**	**10**	**14**-**30**
	**End points**								
	**Prescreen**	**Screen Visit 1**	**Screen Visit 2**	**Baseline WK −6**	**WK −5 to WK −1**	**WK 0 Quit Week**	**WK 2 Follow-up**	**WK 4 Follow-up**	**WK 8, 10, 16, 24 Follow-up**
**Measures for screening**									
Smoking history		X							
Motivation to quit	X								
ASI-16	X								
Exercise frequency	X								
C-SSRS		X							
SCID-NP		X							
Drug screen		X							
Medical history			X						
Physical exam			X						
Laboratory testing			X						
Maximal exercise testing			X						X
**Measures of post-cessation smoking outcomes**									
Point prevalence abstinence				X	X	X	X	X	X
Timeline follow-back				X		X	X	X	X
Carbon monoxide		X		X	X	X	X	X	X
Saliva cotinine									X
**Measures of putative mediators**									
MWS				X	X	X	X	X	X
ASI-III				X	X	X	X	X	X
IDAS				X	X	X	X	X	X
**Measures of treatment integrity/acceptance**									
Credibility/Expectancy					X				
Therapist adherence					X	X	X	X	X
Patient adherence					X	X	X	X	X
Fitness			X						X
Adverse events					X	X	X	X	X
Concurrent treatment	X	X		X		X	X	X	X

The present manuscript details the STEP protocol. In addition to providing a detailed description of a novel exercise intervention for high AS smokers, this manuscript also provides a rationale for design considerations of the study. Specifically, these include the development of an exercise intervention of appropriate length and intensity, the determination of a comparable control group, the decision to use the nicotine patch, and the selection of meaningful follow-up time points.

## Methods/Design

### Overview

**Figure 2:**
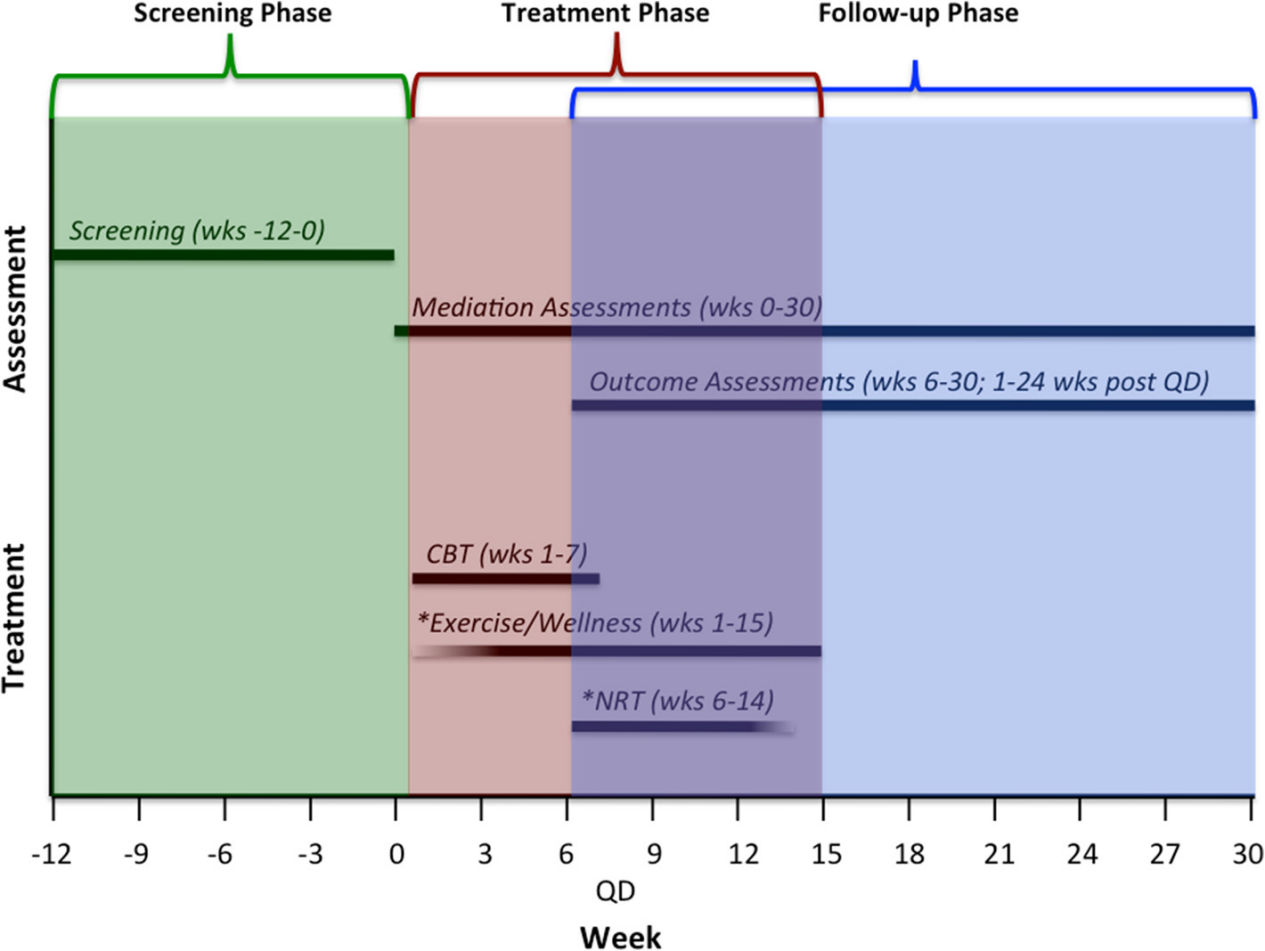
Overview of study flow.

One hundred and fifty eligible participants who reported elevated AS and who are currently smoking will receive the standard treatment (ST) for smoking cessation: cognitive behavioral therapy (CBT) for relapse prevention along with nicotine replacement therapy (NRT). In addition, all participants will be randomized to participate in either an exercise intervention (ST + EX) or a health and wellness education support group (ST + CTRL). As can be seen in Figure 2, all participants will meet three times a week for 15 weeks, receiving CBT once a week for the first 7 weeks, and three exercise or wellness sessions (depending on randomization) per week for the entire 15-week intervention. The participants will be asked to set a quit date for 6 weeks after the baseline visit and smoking cessation outcomes will be measured up to 6 months following the quit date (see Figure 2).

The Institutional Review Board of Southern Methodist University approved the study and a Data Safety and Monitoring Board provides ongoing monitoring. The study is funded by the National Institute of Drug Abuse (NIDA; R01DA027533) and is registered on clinicaltrials.gov (ID: NCT01065506). The STEP study is currently in the recruitment phase.

### Eligibility criteria

Participants will be sedentary men and women with high AS, ages 18 to 65, who have been daily smokers for at least 1 year and are currently smoking an average of at least 10 cigarettes per day. High AS is defined by a score of 20 or greater on the 16-item Anxiety Sensitivity Inventory (that is, above the community norm and a cut-off point shown to be associated with increased risk of future panic and other anxiety problems; [61]). Sedentary is defined as participating in moderate-intensity exercise less than 2 days/week (duration must be 30 min or less each time). Participants must also report a motivation to quit smoking in the next month of at least 5 on a 10-point scale. In addition, results of a physical exam and maximal exercise test conducted by a protocol-approved physician (described in further detail under Assessments) must indicate that no conditions are present that would otherwise be a contraindication of exercise. Participants must also be capable and willing to provide informed consent, attend all study sessions, and adhere to study protocol.

To preserve high internal validity and reduce risk of adverse events, we employ the following exclusion criteria: (1) use of other tobacco products; (2) general medical condition(s) that contraindicate exercise; (3) resting blood pressure of ≥160 systolic and/or 100 diastolic who are not receiving treatment for high blood pressure; (4) blood lipid levels ≥240 mg/dl with LDL-C ≥160 mg/dl or triglyceride levels ≥300 mg/dl (individuals receiving medical treatment for lipid abnormalities with lipid levels above the cut-offs will be eligible with physician written approval); (5) body mass index ≥40; (6) currently suicidal or high suicide risk; (7) current or past psychotic disorders of any type, or co-morbid psychiatric conditions that are relative or absolute contraindications to the use of any treatment option in the study protocol; (8) currently pregnant, planning on becoming pregnant in the next year, or current breastfeeding; (9) abuse or dependence of alcohol, depressants, dissociative anesthetics, hallucinogens, opioids, or cocaine or cannabis dependence within the last 6 months; (10) psychotherapy initiated within the past 3 months, or ongoing psychotherapy of any duration directed specifically toward the treatment of anxiety or mood disorder other than general supportive therapy; and (11) current use of any psychotherapy or pharmacotherapy for smoking cessation not provided by the researchers, including Chantix, Zyban, Welbutrin, and Nortriptyline.

### Screening and randomization

#### Prescreen

An Internet prescreen is conducted to determine potential eligibility for all potential participants who respond or are referred to us via various recruitment strategies. The prescreen is the first point of contact for participants, and it allows us to ask critical information about the potential participant’s willingness and ability to commit to the frequency of clinic visits as well as the assessment of inclusion criteria. The Internet site used to obtain participant information (including both the prescreening and subsequent assessment batteries) is a safe and secure website called Qualtrics. Qualtrics is a sophisticated web survey tool that has been used in experimental research and is designed to ensure the security of data transmission and protection. Qualtrics offers Transport Layer Security (TLS) encryption (HTTPS) and survey security options like password protection and HTTP referrer checking. Qualtrics is a HIPAA compliant company, and servers are stored in a tier one data storage facility that includes adequate security measures.

Persons who appear eligible based on the online prescreen receive a follow-up telephone call to verify prescreen eligibility. Those deemed eligible are invited to come to the study facility for diagnostic and medical screening.

#### Screening visit 1: Psychiatric diagnostic screening

Upon arrival, participants receive an informed consent form explaining the details of the study, potential benefits and risks of participation, and the procedures they will undergo if they choose to participate. After reading the informed consent, a study coordinator discusses these issues with the potential participant and answers any questions he or she has about the study and participation. If the individual chooses to sign the informed consent, he or she begins the psychiatric evaluation.

The psychiatric evaluation begins with the Structured Clinical Interview for DSM-IV Diagnosis of Axis I Disorders Non-Patient Version (SCID-NP) to evaluate the presence psychiatric exclusion criteria. The interview also assesses for primary and secondary diagnoses (if applicable). Participants complete a cheek swab drug test to verify their previously self-reported lack of substance use. Once the initial screening visit is complete, participants are referred to an outside clinic for medical screening and maximal exercise testing.

#### Screening visit 2: Medical screening and maximal exercise testing

The medical examination is used to evaluate potential participants’ eligibility to begin a program of physical activity by determining risk based on exercise guidelines established by the American College of Sports Medicine (ACSM). The examining physician conducts a physical examination that consists of reviewing the participants’ past medical and health history, and their current physical health. Subsequently, the physician orders laboratory testing and complete exercise testing on a treadmill (see below). Based on the information collected during this visit (for example, medical history, physical exam, laboratory testing, exercise testing), the examining physician determines whether the participant meets medical eligibility criteria for the study. Additionally, the data obtained during exercise testing is used to determine (1) the prescribed exercise intensity for participants randomized to the ST+EX condition and (2) pretreatment fitness (functional capacity) as indexed by metabolic equivalents (METs).

#### Baseline/randomization

If deemed eligible after the diagnostic and medical screening visits (based on the inclusion and exclusion criteria presented previously), participants are invited to come in for baseline assessments within 3 weeks. We selected a 3-week window to allow sufficient time to form a cohort/group while also minimizing pre-randomization attrition. Baseline assessments for both conditions include vital signs, height and weight, and a reading of expired air carbon monoxide levels (assessed with a carbon monoxide monitor [62]). Additionally, participants meet their group members, receive an introduction to the study, and are informed of their condition assignment, which takes place after the first group member arrives for the baseline session. The project Biostatistician (DR) oversees the randomization process. Participants are randomized to either the ST+CTRL or ST+EX conditions. Prior to analyzing the data, the project Biostatistician will check the balance of randomization and statistically control for any factors that are imbalanced.

### STEP interventions

#### Standard treatment for smoking cessation (ST)

**Figure 3:**
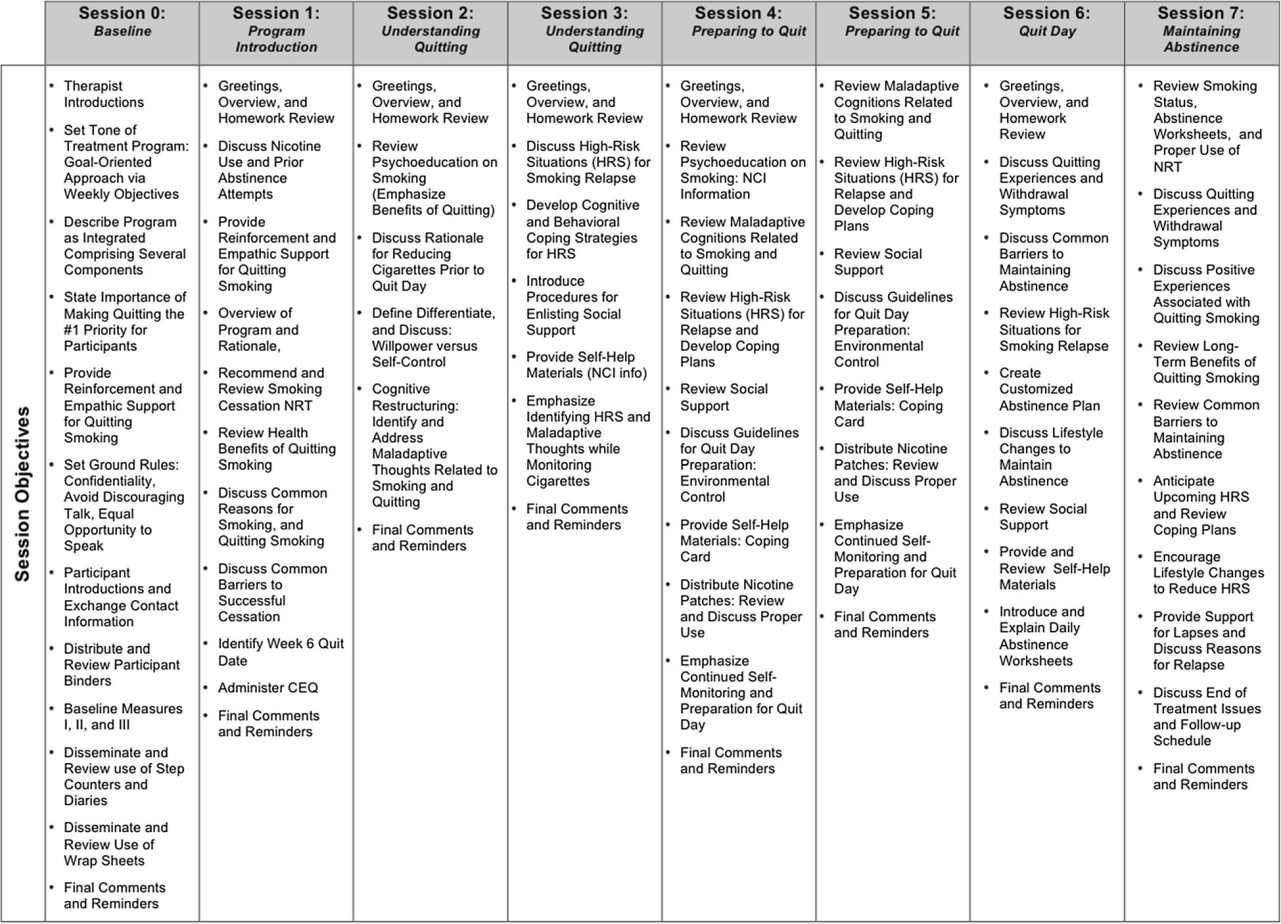
Overview of session objectives.

All participants receive standard treatment for smoking cessation based on the most recent clinical practice guideline from the US Department of Health and Human Services, Treating Tobacco Use and Dependence [2]. This includes CBT for smoking cessation combined with NRT. This standard treatment is delivered through manualized once-weekly 60-min sessions over a 7-week period (Smoking Cessation Program; Therapist Guide [63]; see Figure 3). Topics discussed include identifying high-risk situations for smoking relapse, self-monitoring of cigarette consumption, identifying maladaptive thoughts and behaviors related to the maintenance of smoking patterns, enlisting social support, and developing coping strategies. The first 5 weeks of the intervention serve as planning and preparation weeks. The first day of week 6 of the study is designated as ‘quit day’, at which point participants are asked to make their quit attempt (see Figure 2). At this time, all participants will be given the option to start NRT. Participants are carefully educated about the proper use of the patch prior to quit day and are instructed to apply one patch daily. Based on the number of cigarettes they are smoking prior to quit day, clinicians offer patients a choice of 21-mg patches (indicated for those smoking >10 cigarettes per day), 14-mg patches (indicated for those smoking 10 or less cigarettes per day), or a 7-mg patch (indicated for those smoking <10 cigarettes per day). They gradually taper to a lower dose of the patch every 2 weeks. All participants are asked to taper off the lowest dose of the patch by week 14, at which time the patch is no longer provided. At each session while using the patch, participants are asked about current side effects they may be experiencing. Again, participants can also elect not to use the patch.

#### Exercise intervention (EX)

##### Intervention overview

One week following the baseline session, participants in the exercise condition initiate a 15-week program involving three 45-min exercise sessions each week. During weeks 1 to 7, the first weekly exercise session is completed following the CBT session. During the first session, clinicians provide participants with a rationale for exercise as a treatment for smoking cessation. They are given a model of the role of AS in the maintenance of smoking, and told how interoceptive exposure (exposure to aversive bodily sensations) plays a role in the treatment of AS. Exercise is then introduced as a means of systematic interoceptive exposure. Participants work together with their therapist to develop an exercise training progression schedule which allows participants to gradually increase their intensity across weeks to reach their prescribed dose by week 4 (see Figure 2). By week 4, participant’s target exercise intensity dose is 77% to 85% of their maximum heart rate (HR) achieved during the pretreatment maximal exercise test.

##### Exercise sessions

Trained and CPR-certified staff supervises exercise sessions. During the session, participants wear a HR monitor to confirm that they are training at the targeted exercise intensity. Participants commence the exercise session by performing an active warm-up of 5 min in duration at a progressively increasing speed until the corresponding dose is achieved. Participants then train at their target HR for 25 min. Five minutes after the warm-up (10 min into the exercise session) and every 5 min thereafter, participants are asked to rate their anxiety and/or distress level using the Subjective Units of Disturbance Scale (SUDS) [64]. Additionally, they are also asked their rate of perceived exertion (RPE) [65], speed, inclination of treadmill, and heart rate. At minute 25, participants initiate a 5-min cool down period during which their speed is gradually reduced. At 30 min, the exercise session concludes and participants stretch with the clinician. Occasionally, participants are reminded of and asked to explain the model and rationale for exercise reducing AS (either before or after the exercise session).

#### Wellness education intervention (CTRL)

Similar to the exercise intervention, the wellness education intervention is a 15-week program involving three 45-min sessions each week. The wellness education protocol was modeled after that used in previous studies of exercise for smoking cessation [60,66]. Specifically, during the first session, participants receive a thorough description of the wellness program along with a rationale for the intervention. Participants are given the rationale that, because people who quit smoking often adopt healthy lifestyle changes after smoking, we have opted to include these changes from the onset of the quit attempt to determine whether the addition of these lifestyle changes during a quit attempt has an effect on smoking cessation outcomes. They are also advised that focusing on small wellness goals each week and gaining small successes may carry over into their smoking cessation goals. Wellness education sessions focus on discussions of a variety of healthy lifestyle topics, such as healthy eating, stress and time management, recommended health screenings, and cancer and cardiovascular prevention. The content in each session is delivered using a combination of lectures, videos, handouts, and discussions while allowing participants to set their own realistic wellness goals, which they can gradually incorporate into their lives.

#### Assessment instruments

Table 1 provides an overview of the frequency and timing of administration for the primary assessments instruments that are used for screening, to measure post-cessation smoking outcomes and putative mediators, and to measure treatment integrity and acceptance. A description of each of these measures is provided next.

#### Measures for screening

##### Smoking history

Smoking history is assessed at Prescreen using indices agreed upon by a National Cancer Institute consensus panel [67]. These indices include measurements of brand and nicotine content of cigarette type, rate of smoking, previous quit attempts and duration, household smokers, age of onset of smoking, and more. This instrument has been successfully used in our previous work [43].

##### Anxiety sensitivity

The 16-item Anxiety Sensitivity Index (ASI-16; [25]) is employed to assess sensitivity to and discomfort with physical sensations. The ASI demonstrates good retest reliability and internal consistency, and excellent convergent validity with other established measures [68,69]. This measure is administered at Prescreen as an eligibility assessment.

##### Suicidality

The Columbia Suicide Severity Rating Scale (C-SSRS; [70]) is administered at Screening Visit 1. The C-SSRS is a standardized measure of current and past self-injurious behavior, suicidal intent, and suicidal behaviors, and demonstrates good reliability and validity [70,71].

##### Psychiatric diagnoses

At Screening Visit 1, the Structured Clinical Interview for DSM-IV non-patient version (SCID-NP) [72] is administered to determine diagnostic exclusions and lifetime prevalence of Axis I diagnoses. The SCID is administered by trained doctoral-level interviewers who are supervised by the senior investigators (JAJS and MBP), as has been done successfully in the past (for example, [43,54]).

##### Medical history

The physician obtains medical history, which includes, but is not limited to, medical diagnosis, previous physical examination findings, history of symptoms, orthopedic problems, medication use, exercise history, and family history.

##### Physical exam

The physician completes a physical exam, which includes: height, body weight, apical pulse rate and rhythm, resting blood pressure, auscultation of the lungs, palpation of the cardiac apical impulse, point of maximal impulse, auscultation of the heart, palpation and auscultation of the carotid, abdominal, and femoral arteries, evaluation of the abdomen, palpation and inspection of lower extremities, tests of neurologic function, and inspection of the skin.

##### Laboratory testing

Laboratory testing is performed including hematology (for example, hemoglobin; hematocrit, red blood cell count (RBC); mean corpuscular hemoglobin (MCH), mean corpuscular hemoglobin concentration (MCHC); mean corpuscular volume (MCV), WBC with differential; and platelet count), biochemistry (sodium; potassium; calcium; chloride; glucose; triglycerides; cholesterol (total); protein (total); blood urea nitrogen (BUN); albumin; creatinine; alkaline phosphatase; bilirubin; aspartate aminotransferase (AST); alanine aminotransferase (ALT); and gammaglutamyl transpeptidase (γGT)), and serum pregnancy test (for women).

##### Maximal exercise testing

A maximal exercise test adapted from the Cornell protocol [73] is performed during screening (pretreatment) and at week 16 to examine the participants’ fitness level and obtain an assessment of maximum HR to formulate exercise dose. Though there are many protocols available for exercise testing, we chose the Cornell protocol because we feel it is less of a burden on participants, thus giving us more accurate results. The Cornell protocol is similar to the Bruce protocol; however, the Cornell protocol is divided into smaller and shorter stages and is generally more applicable to patients with limited exercise tolerance because of the smaller workload increments. Prior to the test, a resting ECG is taken and resting HR and blood pressure are measured. Participants then exercise at a rate that gradually increases in speed and incline in 2-min intervals for a maximum of 14 min. Participants are instructed that they may stop exercising at any point, but are encouraged to continue until they reach their maximal exercise dose. Maximal exercise is defined as an inability to continue exercise despite vigorous encouragement. Heart rate is measured continuously by ECG, and blood pressure is monitored every 2 to 3 min by recording brachial artery cuff pressure.

#### Measures of post-cessation smoking outcomes

##### Smoking status

Self-reports of smoking status are collected from participants weekly from quit day through Week 10 post-quit day and at Weeks 16 and 24 post-quit day. Participant reports of abstinence at all assessments are verified by expired carbon monoxide, and additionally with saliva cotinine at the 16- and 24-week interviews (see Table 1). The main outcome analyses are based upon 7-day point prevalence abstinence (that is, reported abstinence of at least 7 days prior to each scheduled follow-up). Self-report is always overridden by objective verification in the conservative direction [74].

In addition to point-prevalence outcomes, we also use the TLFB procedure for assessing the time to first smoking lapse and the time to first relapse, defined as the seventh day on which smoking occurs. The TLFB procedure demonstrates good reliability and validity [75] and we recently validated the TLFB for the assessment of adult cigarette use and to measure smoking among high AS persons over time [76]. The TLFB is administered at each assessment to assess cigarette use since the previous assessment.

##### Biochemical verification

Self-reported abstinence at the 16- and 24-week follow-ups are verified by saliva cotinine (cutoff value of 10 ng/mL) for stated abstinence of 2 weeks or more (cotinine may be incompletely metabolized before this time), and carbon monoxide analysis of breath samples (8 ppm cut-off) for stated abstinence of 24 h to 2 weeks [62]. Saliva samples are frozen for shipment to an outside laboratory, which determines cotinine level by radioimmune assay. Expired air carbon monoxide levels are assessed with a carbon monoxide monitor [62]. Detected values above the stated cutoff scores are considered indicative of smoking.

#### Measures of putative mediators

##### Minnesota withdrawal scale

We monitor withdrawal severity with the Minnesota Withdrawal Scale (MWS), a 10-item scale that is reliable and sensitive [77,78].

##### Anxiety sensitivity index-III

The Anxiety Sensitivity Index-III (ASI-III) is an 18-item measure in which respondents indicate the degree to which they are concerned about possible negative consequences of anxiety-related symptoms [79]. These items were derived from the Anxiety Sensitivity Index (ASI) [25] and the Anxiety Sensitivity Index-Revised (ASI-R) [80]. ASI-III and its subscales show improvements over previous measures of the construct regarding reliability and factorial validity; as well as convergent, discriminant, and criterion-related validity [79].

##### Inventory of depression and anxiety symptoms

The Inventory of Depression and Anxiety Symptoms (IDAS) is a 64-item measure designed to assess specific symptoms of major depression and anxiety disorders [81]. The scales are internally consistent, show excellent convergent validity, and demonstrate good discriminant validity [81].

#### Measures of treatment integrity and acceptance

##### Treatment credibility and expectancy

The Treatment Credibility/Expectancy Questionnaire (CEQ) is a commonly used six-item measure assessing treatment credibility and expectancy [82]. We administer the scale after the first treatment sessions of the ST+CTRL and ST+EX protocols to later examine whether treatment expectancy or credibility varied between the intervention conditions.

##### Therapist adherence

All sessions for CBT and the CTRL interventions are audio-taped and 10% will be rated by independent raters (that is, raters not otherwise associated with the treatment delivery) to assess therapist adherence to and competence with the treatment protocol. Rating checklists and scales were developed for this use.

##### Patient adherence

Patient adherence to the smoking cessation intervention, exercise intervention, and wellness program are assessed by taking attendance at each session (that is, number of total sessions attended). Adherence to the nicotine patch usage is assessed at Weeks 1 to 8 following the quit date. A significant difference in fitness (functional capacity) as expressed in METs between ST+EX and ST+CTRL conditions provides evidence of integrity of the exercise intervention. An assessment team that is blind to study condition will conduct the exercise testing.

##### Adverse events

During the treatment period of the study, the assessment of adverse events occurs at each weekly assessment using standard reporting forms. Participants’ blood pressure (using a brachial artery cuff) and pulse are measured at baseline and at each weekly assessment

##### Concurrent treatment

Use of medications other than nicotine patch, other aids to smoking cessation, and participation in any concurrent psychotherapeutic treatment are assessed at each major outcome assessment (for example, baseline, quit date, weeks 2, 4, 8, 10, 16, and 24 following quit date) point.

#### Analytic methods

Our analyses of the effects of treatment on point prevalence abstinence (PPA) will be conducted using Generalized Linear Mixed Models (GLMM) because it includes all subjects in the analysis, regardless of whether they have missing data. This approach to analyzing PPA was recommended by a working group formed by the Society for Research on Nicotine and Tobacco [83].

The growth curve of abstinence rates over time will be modeled as quadratic because previous research [60] indicates that abstinence rapidly decreases shortly after quit date, but the rate of decline in abstinence levels off over time. We will focus on treatment differences in the slope of change in PPA because slopes have lower variance and greater reliability than means at particular assessments [84], thus yielding greater power and better generalizability. Secondarily, we will test for differences between treatment conditions at particular assessment points by centering our time variable at that particular assessment.

Cox proportional hazards survival analysis will be employed to examine the extent to which ST+EX increases the latency to first smoking. We expect that ST+EX will be associated with a lower risk of lapsing compared to ST+CTRL. Also, we expect mean time to smoking relapse (defined as the seventh occurrence of smoking after quit date) to be longer for those assigned to ST+EX than those assigned to ST+CTRL.

The paths in the mediation model shown in Figure 1 will be calculated using multilevel models (MLM) to assess the ‘a’ paths and GLMM to assess the ‘b’ paths. Each indirect, mediated pathway (for example, the effect of treatment on abstinence that is mediated by AS, path a_3_*b_3_) will be tested for significance using bias-corrected bootstrapping [85]. We expect each mediated pathway to be moderated by exercise condition. Thus, we will follow the procedures suggested by Tein and colleagues [86] to calculate the size of the mediated pathway for each treatment condition separately.

##### Treatment dose

Because some of our participants will not attend all treatment sessions, we will include number of treatment sessions received as an additional predictor in our major analyses. If treatment dose has a significant impact on outcomes, we will use the procedures suggested by Aiken and West [87] to determine the level of dosage necessary for a significant treatment effect on abstinence.

##### Missing data

Following the suggestions of Hall and colleagues [83], we will rerun our primary analyses including dummy codes for various missing data patterns (for example, no missing data, sporadic missing, or all missing after a certain time point) to determine if missingness impacts our findings and if the differences between ST+EX and ST+CTRL depend on the missing data pattern (that is, pattern mixture modeling [88]).

##### Power analyses

We performed Monte Carlo simulations to estimate the sample size necessary to detect hypothesized effect sizes in our analyses. Since the mediation analysis has the lowest power, sample size estimates were based on that analysis. We assumed that we would obtain an average of 57% (4 out of 7) of the assessments from the participants. Our Monte Carlo analysis performed 500 simulations, with each simulation computing a bias-corrected bootstrapping analysis of the mediated effects using 1,200 resamples. Results indicate that it would take a sample size of 150 to have a power of 0.80 to detect significant mediated effect if the ‘a’ path has a large effect size and the ‘b’ path has a medium effect size (see Figure 1). Thus, our target sample size was set at 150.

Next, we determined the power for our primary hypothesis that PPA at the 6-month follow-up would be higher in ST+EX than in ST+CTRL. Given the sample size of 150 determined by our mediation power analysis, we calculated the smallest PPA that we could detect with power >0.80. Assuming that the PPA in ST+CTRL will be 20% [60], our Monte Carlo simulations indicate that we will be able to detect a significant difference between ST+EX and ST+CTRL with power >0.80 if the PPA rate in ST+EX is 35% or greater.

### Rationale for key study protocol choices

#### Sample

A key decision was the selection of participants high in AS as opposed to some other anxiety or mood-related risk factor, because of the specific prediction offered by AS across multiple studies (see introduction). High AS is operationalized by an ASI-16 score of ≥20. Also, we chose to study adult daily smokers who are currently motivated to quit because our intervention will not involve any modules (for example, motivational interviewing) designed to increase motivation for cessation. Smokers using other types of nicotine products (for example, smokeless tobacco, cigars) will not be included in the sample, as the study was designed specifically for reducing cigarette smoking. Assessments of cigarette smoking could be confounded by inclusion of other types of nicotine products.

#### Inclusion of nicotine patch in standard treatment

Because recent clinical guidelines recommend that all smokers attempting to quit smoking receive pharmacotherapy [89,90], we believe it is important to make pharmacotherapy available as part of our standard smoking cessation treatment. We selected the transdermal nicotine patch (TNP) because of the extensive empirical literature supporting its effectiveness and safety, its ease of use, and its relatively benign side effect profile, which have led to its approval as an over-the-counter medication. We chose to provide the patch for 8 weeks because use of TNP for longer than 8 weeks does not appear to improve treatment efficacy [91]. Overall, using TNP with standard smoking counseling allows us to examine if the ST+EX is more efficacious than existing (standard) treatment. Because TNP is optional, its use will be tracked for each study participant.

#### Dose, duration, and format of the exercise intervention

We elected to use the public health recommended dose of vigorous-intensity exercise (that is, 75 min of aerobic exercise at >76% of maximum heart rate per week [2]; because: (1) research suggests that it can yield large reductions in AS [54]; and (2) a comparable dose was employed in the [60] study that demonstrated the efficacy of exercise as an aid for smoking cessation. In order to ensure that the exercise intervention is of sufficient duration [57], we decided to match the length of the exercise intervention (that is, 15 weeks) to that of established psychotherapies that show clear efficacy for reducing AS, depressed mood, and anxiety among individuals with elevated levels of AS (for example, cognitive-behavioral treatment of panic disorder; for example, [92-94]). Consistent with the American College of Sports Medicine (ACSM) exercise prescription guidelines [95], participants are encouraged to progress to the study dose during the first 3 weeks (by varying intensity and duration of exercise) of the smoking cessation program. From week 4 (that is, 2 weeks prior to the quit date; see Table 1) to week 15, participants are expected to exercise at the study dose. Finally, participants are required to complete their exercise sessions at the study site’s exercise facility under the supervision of a trained staff member. Our decision to utilize a supervised instead of a combination supervised/home-based format, which may be more attractive from a cost-efficacy perspective, is guided by the specific aims of our study. Specifically, in order to examine the efficacy and mechanisms of exercise for smoking cessation, it is essential to achieve the highest intervention adherence rates possible, which is more easily accomplished using a supervised instead of a combination supervised/home-based format (for example, [60,66]).

#### Inclusion of a control condition

In order to isolate the effects of the exercise intervention, while at the same time reducing the risk of differential attrition, we needed to equate the two conditions for common factors (for example, expectancy effects, demand characteristics, social contact, expert contact). We are accomplishing this by requiring participants who are not assigned to receive the exercise intervention to attend a 15-week Wellness Program. The Wellness Program was developed and previously implemented effectively by our team (for example, [60,66]). Matching the exercise intervention for contact time, participants attend three 45-min sessions each week. Each session (for example, lectures, handouts, films, and discussions) focuses on lifestyle issues, such as healthy eating and prevention of cancer and cardiovascular disease.

#### Using a 6-month over a 12-month follow-up

We had several reasons for choosing a 6-month post-quit follow-up over a 12-month follow-up. First, a 6-month follow-up seems reasonable given the well-accepted observation that most smoking relapses occur in the first 3 months post-cessation [89,90]. Second, we want to examine point prevalence abstinence at a number of key time points of interest: 2, 4, 8, 10, 16, and 24 weeks following quit date (see Figure 2 and Table 1). The first two time points reflect our particular interest in immediate vulnerability to relapse as a result of elevated anxiety symptoms. Week 2 also corresponds to the end of the CBT intervention. Week 8 corresponds to the end of nicotine patch usage, Week 10 corresponds to the end of the EX/CTRL interventions, and Weeks 16 and 24 allow for longer-term follow-up. However, it should be noted that we also employ the smoking TLFB interview, which provides information on daily smoking rates over the 6-month follow-up. We previously demonstrated the reliability and validity of the TLFB method for use in assessing adult smoking outcomes [63].

## Discussion

The primary objective of the STEP study is to contribute to the growing body of literature calling for the development of specialized smoking cessation interventions for anxiety vulnerable individuals. If effective, the use of exercise as a component of treatment for smoking cessation would have a significant public health impact. In addition to improving smoking cessation treatment outcome, exercise is expected to offer benefits to overall health, which may be particularly important for smokers. Further, the proposed study (that is, design, assessment schedule, and analyses) offers the potential to improve the understanding of the mechanisms that underlie these effects. The specific design considerations of the exercise intervention provide a dose and intensity that is thought to enact beneficial biological and psychological changes while also allowing for participant satisfaction and flexibility. The utilization of a wellness education group provides an adequate, time-matched control necessary in order to examine the specific effects of exercise.

The anticipated benefits of the study are twofold: (1) the results will yield knowledge about the combined effects of exercise with standard smoking cessation treatment; and (2) the results will advance understanding of the factors related to anxiety and relapse to smoking after attempts at smoking cessation. The results of the study may also inform strategies to match treatments to specific characteristics of smokers. Lastly, data yielded by this study is expected to guide future studies that aim to determine the optimal dose and intensity for exercise-based smoking cessation interventions as well strategies to enhance motivation to exercise and motivation to quit smoking.

### Trial status

The trial is currently in the recruitment phase.

## Abbreviations

ACSM: American College of Sports Medicine; AS: Anxiety sensitivity; CBT: Cognitive behavioral therapy; NRT: Nicotine replacement therapy; PPA: Point prevalence abstinence; ST: Standard treatment; ST+CTRL: Standard treatment plus control condition; ST+EX: Standard treatment plus exercise condition; STEP: Smoking Termination Enhancement Project; TLFB: Timeline follow back.

## Competing interests

Drs Smits and Otto receive royalties from Oxford University Press for books on exercise for mood and anxiety disorders. All other authors declare that they have no competing interests.

## Authors' contributions

JAJS and MJZ conceived the study and participated in its design and coordination. DR, BHM, TSC, GMF, MBP, and MWO contributed to the design of the study and provided consultation and supervision. MLD, LBD, and NFB participated in the coordination of the study. All authors contributed to the drafting of the manuscript and have read and approved the final manuscript.
